# Left ventricular geometric patterns and adaptations to hemodynamics are similar in elderly men and women

**DOI:** 10.1186/1471-2261-11-25

**Published:** 2011-05-27

**Authors:** Said Masiha, Johan Sundström, Lars Lind

**Affiliations:** 1 Department of Cardiology, Uppsala University Hospital, Uppsala, Sweden; 2 Department of Internal Medicine, Uppsala University Hospital, Uppsala, Sweden; 3 Department of Internal Medicine, Uppsala University Hospital, Uppsala, Sweden

**Keywords:** Gender, Left ventricular geometry, left ventricular hypertrophy, echocardiography, Doppler

## Abstract

**Background:**

Common conditions such as obesity and hypertension result in hemodynamic alterations that will induce remodeling of the left ventricle (LV). However, differences between the genders in the relationship of hemodynamics to LV geometry are not well known.

The present study aims to investigate differences between the genders in this respect, in a sample of elderly persons.

**Methods:**

Echocardiography and Doppler was performed in a population-based sample aged 70 - The Prospective Investigation of the Vasculature in Uppsala Seniors (PIVUS) study (n = 922).

Hemodynamic patterns obtained by echocardiography and Doppler were evaluated in relation to four LV geometric groups (normal, concentric remodeling, eccentric hypertrophy and concentric hypertrophy).

**Results:**

No significant difference between the genders was observed regarding the prevalence of the LV geometric groups.

Mean values of most evaluated echocardiography and Doppler variables differed between men and women, such as LA, IVS, LVEDD and IVRT, but the relationship of hemodynamic variables to LV geometric groups did not differ between the genders.

**Conclusions:**

Although mean values of many echocardiographic variables differed between men and women, the LV geometric adaptations to a given hemodynamic load appear similar in both genders.

## Background

Alteration of the left ventricle (LV) as a consequence of hypertension is a well known phenomenon and numerous studies have demonstrated that LV hypertrophy is a strong and independent predictor of cardiovascular events [1-4].

Several studies have demonstrated the impact of hypertension both as isolated systolic hypertension and in combination with diastolic hypertension on LV structures [5-9].

Different patterns of LV geometry as a result of hypertension were first described in a study by Ganau and co-workers in 1992 [10]. That study included 165 hypertensive and 125 middle-aged adults and described LV geometry using a combination of LV mass and the relation of muscle wall thickness to cavity size (relative wall thickness, RWT).

Surprisingly, the study demonstrated that the most common LV hypertrophy (LVH) category was not the classical one, concentric hypertrophy. 52% of the hypertensive patients had a completely normal LV mass and RWT. 13% had a normal LV mass, but increased RWT and were classified as concentric remodeling. Of the remaining persons 27% had an eccentric LV hypertrophy and only 8% had concentric LVH. Furthermore, it was shown that different types of LV geometrical adaptations were associated with different hemodynamic patterns. A 10-year follow-up of 253 persons with initially uncomplicated essential hypertension revealed that persons with concentric LV geometry had the highest risk for cardiovascular death (31%) and morbid events (11%). The lowest risk was observed in the group with normal LV geometry (no cardiovascular event and 11% risk for morbid events) [10].

Andrén and co-workers investigated a population-based sample of 584 elderly men [11]. Alterations in LV geometry were observed in both elderly men and in those with hypertension or coronary heart disease, but were more pronounced in the latter group. The prevalence of persons with normal as opposed to hypertrophic LV was different in these two studies.

In addition to different hemodynamic patterns the geometric groups often have different clinical backgrounds. Persons with coronary heart disease and cardiomyopathy tend to have a higher prevalence of eccentric LVH. Concentric LVH is observed more often in persons with hypertension or valvular disease (aortic stenosis) and patients with diabetes or metabolic syndrome tend to have a higher prevalence of concentric remodeling [12,13]

A study by Krumholz and co-workers demonstrated the association of isolated hypertension with increased left ventricular mass in men and women, the geometric pattern of increased LV mass differed by sex. Women demonstrated mainly a pattern of concentric hypertrophy while an eccentric pattern mainly was observed in men [14].

Thus, few studies have reported gender differences in proportions of LV geometric patterns in the general population. Our primary aim in the present study was therefore to study gender differences in the prevalence of LV geometric groups. A secondary aim was to investigate differences between genders regarding hemodynamic patterns in different LV geometric groups.

## Methods

### Subjects

Eligible were all subjects aged 70 living in the community of Uppsala, Sweden. The subjects were randomly chosen from the register of community living and invited shortly following their 70^th ^birthday. 1016 subjects participated giving a participation rate of 50.1%. The study was approved by the Ethics Committee of the University of Uppsala. Signed informed consent was given by all subjects.

Approximately 10% of the cohort reported a history of coronary heart disease, 4% reported stroke and 9% diabetes mellitus. Almost half the cohort reported any cardiovascular medication (45%), with antihypertensive medication being the most prevalent (32%). Fifteen percent reported use of statins, while insulin and oral antiglycemic drugs were reported in 2 and 6%, respectively.

In the sample, 22% were on beta-blocking drugs, 11% on calcium antagonists, 12% were on diuretics, 8% were taking ACE-inhibitors and 8% were on regular medication with an ARB.

### Basic investigations

Prior to their examination, subjects completed a questionnaire concerning their medical history, regular medication and smoking habits.

All subjects were investigated in the morning after an overnight fast and no medication was taken on the day of the investigation. Participants were in the supine position in a quiet room at constant temperature.

Recordings of height, weight and abdominal and hip dimensions were performed and blood samples were taken and analysed by standard laboratory techniques.

### Echocardiagraphy and Doppler

A comprehensive two-dimensional and Doppler echocardiography was performed with an Acuson XP124 cardiac ultrasound unit (Acuson, California, USA) A 2.5 MHz transducer was used for the majority of the examinations. Presence of stenosis or regurgitation in the mitral and aortic valves was recorded by use of colour and continuous Doppler. A complete echocardiographic examination was only successfully performed in 922 due to technical problems to obtain images of good quality in 94 subjects.

LV dimensions were measured with M-mode on-line from the parasternal projections, using a leading edge to leading edge convention. Measurements included left atrial diameter (LA), interventricular septal thickness (IVS), posterior wall thickness (PW), left ventricular diameter in end diastole and end systole (LVEDD, LVESD). Left ventricular relative wall thickness (RWT) was calculated as (IVS+PW)/LVEDD. Echocardiograms were read on-line. M-mode recordings were used for calculations of LV dimensions, such as LVEDD, LVESD, IVS and PW, being the basis for calculations of LV mass.

Left ventricular mass (LVM) was determined from the Penn conversion. LVM was then indexed for height^2.7 ^to obtain left ventricular mass index (LVMI).

LV geometry was also divided into 4 categories according to Ganau et al. A normal LV geometry was considered to be present if LVMI was normal (< = 51 g/m2.7) and relative wall thickness (RWT) < 0.45 Concentric LVH was defined as LVMI above the threshold for LVH together with RWT > = 0.45, but if RWT was below this cut-off for RWT eccentric LVH was present. If LVMI was normal but RWT > = 0.45 the LV geometry was denoted as concentric remodeling.

Left ventricular volumes were calculated according to the Teichholz formula 7*D3/(2.4+D) and from that value ejection fraction (EF) was calculated.

The left ventricular diastolic filling pattern of the mitral inflow was obtained from the apical transducer position with the pulsed Doppler sample volume between the tips of the mitral leaflets during diastole. The peak velocity of the early rapid filling wave (E wave) and the peak velocity of atrial filling (A wave) were recorded and the E to A ratio (E/A) was calculated. Left ventricular isovolumetric relaxation time (IVRT) was measured as the time between aortic valve closure and the start of mitral flow using the Doppler signal from the area between the left ventricular outflow tract and mitral flow. Presence of a restrictive filling pattern was evaluated in subjects with a reduced EF. This pattern was considered to be present if E/A-ratio was >1.5 and IVRT < 96.

Also the time between the A and the next E-wave was measured (A-E) together with the ejection time (ET, interval of flow in the left ventricular outflow tract). The myocardial performance index (MPI, also called the Thei-index) was calculated as: A-E minus ET divided by ET. MPI was not included in the original protocol and was therefore only measured in the last 852 consecutive subjects.

Subjects with significant valvular disease were excluded from the analysis (n = 5).

### Statistical Analysis

StatView (SAS inc. NC; USA) was used for statistical analysis. If variables failed to show normal distribution (the E/A-ratio) a natural logarithmic transformation was done. Analysis of Variance (ANOVA) was used to compare different groups and Analysis of Covariance (ANCOVA) was used for correction for gender and history of myocardial infarction. Any post-hoc analysis was carried out with Bonferroni correction. Chi-square analysis was used to relate two nominal variables. Only p-values < 0.05 were accepted as statistically significant.

## Results

There were no significant differences regarding the frequency of antihypertensive treatments between the genders. Systolic blood pressure and heart rate differed significantly between the genders but there was no significant difference regarding diastolic pressure.

The echocardiographic measurements indicating different geometries such as IVS, PW, LVEDD and LVESD were significantly larger in men (p < 0.0001) but on the other hand no significant difference could be demonstrated regarding RWT, LVMI, CI or TPRI between men and women.

Among variables evaluating LV diastolic function, there was no significant difference in E/A-ratio (p < 0.58) between the genders but IVRT and ET were significantly shorter in men (p < 0.0001) (table 1).

**Table 1:** Mean values ± SD for echocardiographic variables in men vs. women

*Echo-variables*	*Men Mean ± SD*	*Women Mean ± SD*	*P-Value*
*N*	460	462	
Hypertensive treatment (%)	31	31	0.99
Hospitalized MI (%)	11.0	3.0	<0.0001
Diabetes (%)	13.3	8.4	0.080
CHF (%)	5.0	3.0	0.19
Manual SBP (mmhg)	146 ± 22	153 ± 23	<0.0001
Manual DBP (mmhg)	79 ± 10	78 ± 10	0.43
BMI (kg/m2)	27.0 ± 3.6	27.0 ± 4.5	0.74
Heart rate (beats/min)	60 ± 8.8	63 ± 8.6	<0.0001
LA (mm)	42 ± 7	37 ± 6	<0.0001
IVS (mm)	11.5 ± 2	10.5 ± 2	<0.0001
LVEDD (mm)	49 ± 5	45 ± 5	<0.0001
PW (mm)	10 ± 1.6	9 ± 1.5	<0.0001
LVESD (mm)	27 ± 6	24 ± 5	<0.0001
E (cm/s)	61 ± 13.5	67 ± 15.6	<0.0001
A (cm/s)	66 ± 15	72 ± 16	<0.0001
IVRT (ms)	124 ± 22	118 ± 20	<0.0001
ET (ms)	270 ± 20	278 ± 20	<0.0001
E/A-ratio	0.97 ± 0.30	0.95 ± 0.25	0.58
E/A-ratio (median, IQR)	0.91 (0.78-1.09)	0.93 (0.77-1.09)	
MPI	0.63 ± 0.17	0.58 ± 0.15	<0.0001
EF	0.65 ± 0.09	0.68 ± 0.07	0.0008
RWT	0.44 ± 0.09	0.44 ± 0.09	0.65
LVMI m^2.7^	44 ± 13	42 ± 13	0.55
CI (Doppler) l/min/m^2^	2.6 ± 0.68	2.6 ± 0.68	0.30
TPRI (Doppler)dynes s cm ^-5 ^m^2^	3264 ± 1009	3222 ± 959	0.56

CHF = Congestive Heart Failure, SBP = Systolic blood pressure, DBP = Diastolic blood pressure, LA = Left atrium diameter, IVS = Interventricular septal thickness, LVEDD = Left ventricular end-diastolic diameter, PW = Posterior wall, LVESD = Left ventricular end-systolic diameter, E = Peak transmitral flow velocity during early diastole, A = Peak transmitral flow velocity during atrial contraction, IVRT = Isovulemic relaxation time, ET = Aortic ejection time, MPI = Myocardial performance index, EF = Ejection fraction, RWT = Related wall thickness, LVMIm^2.7 ^= Left ventricle mass index for height^2.7^, CI = Cardiac index, TPRI = Total peripheral resistance index

**Figure 1:**
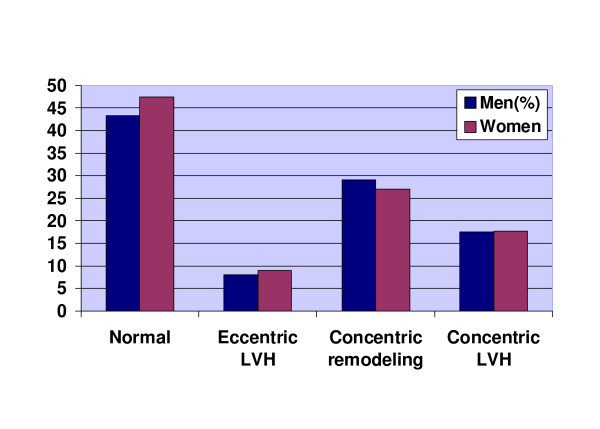
**Frequency distribution of the four different left ventricular geometrical groups in men vs. women **LVH = Left ventricular hypertrophy, P = 0.50 for a gender difference for LV-geometry groups (χ^2^-test).

The frequency distribution of the LV geometric groups indicated no significant differences between the genders (P-value 0.50) (Figure 1).

Since there were no significant interactions between gender and the LV geometric groups (table 2, interaction p-values) regarding the hemodynamic patterns, except for systolic blood pressure, the genders were analyzed together in the following section. Systolic blood pressure was significantly higher in women than men in all LV groups except eccentric hypertrophy where levels did not differ significantly.

**Table 2:** Mean values ± SD for echocardiographic hemodaynamic variables evaluating differences between LV-geometrical groups.

Echo-variables	Normal	Eccentric LVH	Concentric remodeling	Concentric LVH	ANOVA P-Value	Interaction P-value
***n***	***416***	***80***	***260***	***162***		
Manual SBP (mmhg)	142 ± 20	154.5 ± 22 ****	151 ± 22 ****	162 ± 24 ****	<0.0001	0.009
Manual DBP (mmhg)	77 ± 10	80 ± 10	79 ± 10	82 ± 11 ****	<0.0001	0.19
E/A-Ratio	0.99 ± 0.25	0.87 ± 0.27 ***	0.96 ± 0.25	0.88 ± 0.20 ****	<0.0001	0.40
ET (ms)	276 ± 21	269 ± 18 *	274 ± 19	275 ± 20	0.15	0.22
MPI	0.57 ± 0.16	0.69 ± 0.22 ****	0.60 ± 0.14	0.66 ± 0.16 ****	<0.0001	0.94
EF(%)	0.67 ± 0.07	0.61 ± 0.11 ****	0.67 ± 0.08	0.67 ± 0.08	<0.0001	0.39
E (cm/s)	64 ± 14.4	60 ± 13.5 *	64 ± 14.6	63 ± 15	0.17	0.75
A (cm/s)	67 ± 15	72.6 ± 17.5 **	69 ± 15	73.5 ± 15 ****	<0.0001	0.47
IVRT (ms)	114 ± 18.6	134 ± 22 ****	124 ± 19 ****	134 ± 21 ****	<0.0001	0.69
CI (l/min/m^2^)	2.7 ± 0.59	3.4 ± 0.63 ****	2.2 ± 0.53 ****	2.7 ± 0.69	<0.0001	0.59
TPRI (Doppler) dynes s cm^-5 ^m^2^	2947 ± 697	2519 ± 512 ***	3946 ± 1112 ****	3228 ± 939 **	<0.0001	0.80
HR	61. 6 62 ± 8.4	61 ± 8.0	62 ± 9.3	61 ± 9.4	0.60	0.61

At right a P-value for a gender-Lv group interaction is given. SBP = Systolic blood pressure, DBP = Diastolic blood pressure, A = Peak transmitral flow velocity during atrial contraction, IVRT = Isovolemic relaxation time, ET = Aortic ejection time, MPI = Myocardial performance index, EF = Ejection fraction, CI = Cardiac index, TPRI = Total peripheral resistance index.

* = P-value < 0.05, ** = P-value < 0.01, *** = P-value < 0.001, **** = P-value < 0.0001 vs normal geometry

Both systolic and diastolic blood pressures were significantly different between the groups. Systolic blood pressure was elevated in all pathological groups, but diastolic blood pressure was significantly increased only in concentric LVH group.

The measurements which are crucial for the division into the geometric groups (RWT, LVEDD, IVS, and PW) were significantly different in the pathological groups compared with the group with normal LV geometry (p < 0.0001).

LV diastolic function indicators such as E/A-ratio and IVRT were significantly pathological in the abnormal LV geometrical groups (p < 0.0001) with exception for E/A-ratio in the concentric remodeling group compared with normal LV geometry. Even ET was not significantly different between the groups (p = 0.15) (table 2).

There was a highly significant difference between the groups concerning systolic hemodynamic values (CI, MPI and EF) (p < 0.0001). EF was lowest in the eccentric LVH group. There was no significant difference regarding MPI in the concentric remodeling group in comparison with normal LV geometry (table 3).

**Table 3:** Mean values ± SD for echocardiographic LV-dimensional variables evaluating differences between LV-geometrical groups

Echo-variables	Normal	Eccentric LVH	Concentric remodeling	Concentric LVH	ANOVA P-value	Interaction P-value
*N*	416	80	260	162		
LA (mm)	37 + 6	43.7 + 6.6 ****	38.5 + 6.5 **	43.2 + 6.0 ****	<0.0001	0.59
LVMI m2.7	35 + 7.4	60 + 9.0 ****	40 + 7.4 ****	61 + 9.2 ****	<0.0001	0.94
IVS (mm)	9.4 + 1.3	11 + 1.2 ****	11.8 + 1.5 ****	13.6 + 1.3 ****	<0.0001	0.91
LVEDD (mm)	47 + 4.5	56 + 5.0 ****	43 + 4.0 ****	49 + 4.3 ***	<0.0001	0.73
PW (mm)	8.5 + 1.0	10.2 + 1.0 ****	10.2 + 1.2 ****	11.6 + 1.4 ****	<0.0001	0.48
RWT	0.38 + 0.04	0.39 + 0.04 *	0.52 + 0.07 ****	0.52 + 0.06 ****	<0.0001	0.78

At right the P-value for a gender-LVgroup interaction is given LA = Left atrium diameter, LVMI m^2.7 ^= Left ventricle mass index for height^2.7^, IVS = Interventricular septal thickness, LVEDD = Left ventricular end-diastolic diameter, PW = Posterior wall, RWT = Relative wall thickness, * = P-value < 0.05, ** = P-value < 0.01, *** = P-value < 0.001, **** = P-value < 0.0001 vs normal geometry

In order to evaluate any particular interaction between hemodynamics and gender regarding LVMI and RWT, we performed two different regression models. In the first, LVMI was the dependent variable and the hemodynamic variables CI, SBP, DBP and TPRI were independent variables together with gender. In this model CI, SBP and gender were significant independent predictors of LVMI (p < 0.0001 for all). In a second step the interaction terms between gender and CI, as well as between gender and SBP, were investigated, but none of them were significant in the model (p = 0.48 and 0.40, respectively).

In the next regression model, LVMI was substituted with RWT as the dependent variable. In this case, SBP and TPRI were the significant independent predictors of RWT (p < 0.0001 for both). However, neither in this case the interaction terms between gender and SBP or between gender and TPRI were significant (p = 0.11 and 0.51, respectively). Thus, taken together, the influence of the hemodynamic factors; CI, SBP, DBP and TPRI on LVMI or RWT were not found to be significantly different between men and women.

In a secondary analysis of the data excluding the 129 subjects with a history of ischemic heart disease, still no significant difference was seen between men and women regarding the LV geometric pattern (p = 0.66). For the results presented in table 2, the differences between the groups regarding echocardiographic and blood pressure differences between the LV groups, still no significant interaction was seen except for SBP. The same was found for the differences presented in table 3.

## Discussion

In clinical practice there is a tendency to consider women`s hearts different from men`s hearts regarding cardiovascular risk and diseases. It is therefore relevant to study whether there are any significant differences between the genders regarding the adaptation of the LV to the different hemodynamic patterns.

### LV geometry patterns

The relation between left ventricle geometry and hemodynamic conditions has previously been studied [10]. The LV geometrical alteration as a result of adaptation to changed hemodynamic conditions was grouped into three different hemodynamic categories.

The same geometrical categories were identified in a study by Andrén and co-workers [11]. As many as 16% of the healthy persons demonstrated presence of left ventricle hypertrophy (mainly eccentric LV hypertrophy). LV hypertrophy was as expected more frequent in the persons suffering from hypertension or heart diseases. Despite the lower blood pressure in that study compared with the study performed by Ganau [10], the proportion of the normal LV geometry was lower and the proportion of persons with LVH was higher. A reasonable explanation was the higher age, longer duration of hypertension but also gender differences, as the Andrén study only included men [11].

In the LIFE study [15], a large series of middle-aged and elderly patients with moderate hypertension and target organ damage were investigated and among those who underwent echocardiography 19% had normal LV geometry, 11% concentric remodeling, 47% eccentric LV hypertrophy and 24% concentric hypertrophy. There were no significant differences among LV geometric subgroups with regard to BP, prevalence of diabetes or peripheral vascular disease. The prevalence of coronary and cardiovascular disease was almost twice as high in patients with concentric LV hypertrophy as in the other subgroups.

A subgroup study of the Framingham heart study [16] demonstrated that persons with concentric LVH had the worst prognosis, followed by those with eccentric hypertrophy, concentric remodeling and normal geometry. Adjustment for LV mass eliminated any association between LV geometry and outcome in women. In men however, there remained a tendency toward an increased risk for all-cause mortality in each group with abnormal LV geometry compared with the normal group.

Obesity has been associated with eccentric LVH, while hypertension has been associated with concentric LVH [17]. Persons with concurrent obesity and hypertension presented a further increase of LVM and wall thickness above values in the merely obese or hypertensive persons and had more frequently LVH. In all these groups women had a higher frequency of abnormal LV geometry. It was suggested that the hearts of postmenopausal women respond more susceptibly to trophic stimuli. In particular concentric LVH was a common finding in women.

A study by Sveälv and co-workers in 2005 demonstrated a significant age-related decrease in end-diastolic and end-systolic volumes which was explained by shortening of the long axis length which resulted in an increased sphericity with age. This remodeling during normal aging appeared to be more pronounced in females [18].

Most of these studies were designed to study the hemodynamic patterns and the remodeling of the left ventricle, but whether there were gender-related differences was not investigated. One of the exceptions was the study by Krumholz and co-workers in which members of the Framingham Heart study and Framingham Offspring Study were investigated [14]. They found that the geometric pattern of LV hypertrophy differed by sex. The subjects of this study were free of clinically apparent cardiovascular disease, not taking antihypertensive medication and without diastolic hypertension.

However we could not find such a difference between the genders in our analysis, probably depending on that our participants were elderly subjects in a general population treated with a great number of CV medications.

In the present study we investigated the genders separately and tried to reveal if there were any significant hemodynamic differences between the LV geometrical categories recognized in the earlier studies. Studying the frequency distribution of the four distinguished LV geometrical groups in men vs. women indicated no significant differences. Almost 43 percent of the men and 47 percent of the women had a normal geometry. The most common abnormal geometry was the concentric remodeling group (28% in males and 27% in females) followed by concentric and eccentric LVH.

Thus, the present study differed from Andrén's study [13] in 70-year old males in that concentric LVH was more common than eccentric LVH. The fact that LV mass was indexed for height in the present study and BSA in Andrén's study could hardly explain this discrepancy, since the subdivision of LVH is highly dependent of RWT, using the same cut-off limit in both studies. Since both studies were conducted in the same town in subjects with the same age and with a similar protocol, the most obvious reason for the discrepancy is that women were included in the present study. However, since no gender differences were seen between the geometric groups in the present study, other yet undiscovered differences must exist.

A significant interaction between gender and SBP was found regarding LV geometry groups. However, an increased SBP were seen in women in all LV geometric groups except the eccentric LVH group. This finding does not indicate that women have a different sensitivity to the SBP level for the development of an abnormal LV geometry, since women in the normal LV group also had an elevated systolic blood pressure.

The proportion of males and females taking antihypertensive drugs were very similar. However, the systolic blood pressure was significantly higher in woman than in men both in the group on antihypertensive treatment, as well as in the group without any treatment. Therefore, the gender difference does not seem to be due to any difference in blood pressure medication. In this age-group, SBP seems to be generally higher in women than in men. The reason for this is not known, but it is clear that this increased SBP in women is not reflected in any higher prevalence of LVH.

### Evaluation of systolic function

Ganau and co-workers presented different hemodynamic patterns in the different LV geometrical categories [10]. Concentric LVH was accompanied with an increased afterload and a normal systolic function. Eccentric LVH associated with a more spherical chamber cavity and increased diameter is a result of increased preload increasing CI. Concentric remodeling with an increased RWT, reduced LV cavity and more elliptic LV chamber shape and normal LVM was caused by an increased afterload and decreased preload resulting in a low CI. Andrén and co-workers showed the same results when measuring CI and TPRI by the Teicholz formula, but in this study the eccentric group was accompanied by a lower EF, AV-plane-displacement and a higher LV-wall motion score indicating an impaired contraction [11]. The hemodynamic patterns in subjects with eccentric and concentric LVH were in this study more like the ones found in Andren's study compared with the study by Ganau et al.

Comparing systolic indices, such as EF and CI in men vs. women indicated a significant discrepancy between the genders regarding EF, but no significant difference in CI. However, despite these differences in EF between the genders, possibly due to an increased prevalence of MI in the males, no significant interactions were seen between gender and LV geometry groups regarding LV systolic variables.

### Evaluation of diastolic function

Andrén and co-workers showed a significant prolongation of IVRT only in the eccentric LVH group and a prolongation of the deceleration time in the concentric LVH group suggesting abnormal relaxation and low LV compliance [11]. Left atrium size was significantly enlarged in both hypertrophy groups as evidence of diastolic dysfunction. E/A-ratio indicated no significant difference between the groups with hypertrophic and normal geometry.

In the present study, the E/A-ratio was significantly lower, LA diameter increased and IVRT longer in all three abnormal categories compared with the normal group, indicating a more severe LV diastolic dysfunction in the present study as compared to the study by Andren et al.

Concerning diastolic variables we found a significant difference in LA size and IVRT, but no significance in the E/A-ratio, between men and women. However, despite this gender difference, no significant interactions were seen between gender and LV geometry regarding LV diastolic function.

### Strengths and limitations of the study

The present sample is an extensive study which investigated more than 1000 individuals consisting of almost the same number of males and females, but it is limited to Caucasians aged 70. So, caution should be made in drawing conclusions about other ethnic and age groups. The present study had a moderate participation rate. However, an analysis of non-participants showed the present sample to be fairly representative of the total population regarding most cardiovascular disorders and drug intake.

LV mass and EF were calculated based on M-mode measurements. 3D ultrasound recordings or myocardial MRI might give more appropriate results. Furthermore, since a large number of the subjects were on different CV medications, it cannot be excluded that use of particular types of drugs of combinations thereof might have confounded the results.

## Conclusions

### What is known about this topic?

Alteration of the left ventricle (LV) as a consequence of hypertension is a well known phenomenon and numerous studies have demonstrated that LV hypertrophy is a strong and independent predictor of cardiovascular events.

However, differences between the genders in the relationship of hemodynamics to LV geometry are not well known.

### What this study adds?

➢ Although mean values of many echocardiographic variables differed between men and women, the LV geometric adaptations to a given hemodynamic load appear similar in both genders.

➢ No significant difference between the genders was observed regarding the prevalence of the LV geometric abnormalities.

## Competing interests

The authors declare that they have no competing interests.

## Authors' contributions

SM: analyzed the data and wrote the manuscript; LL: contributed to the study design and organized the study; LL and JS: revised the manuscript critically for important intellectual content. All authors have read and approved the final manuscript.

## Pre-publication history

The pre-publication history for this paper can be accessed here:

http://www.biomedcentral.com/1471-2261/11/25/prepub
